# Assessing structure and disorder prediction tools for *de novo *emerged proteins in the age of machine learning

**DOI:** 10.12688/f1000research.130443.1

**Published:** 2023-03-29

**Authors:** Margaux Aubel, Lars Eicholt, Erich Bornberg-Bauer

**Affiliations:** 1 Institute for Evolution and Bidiversity, University of Muenster, Muenster, 48149, Germany; 2 Department Protein Evolution, Max Planck-Institute for Biology, Tuebingen, 72076, Germany

**Keywords:** de novo proteins, disorder, pLDDT, protein structure, structure predictions, AlphaFold, Natural language models

## Abstract

**Background:** *De novo *protein coding genes emerge from scratch in the non-coding regions of the genome and have, per definition, no homology to other genes. Therefore, their encoded *de novo *proteins belong to the so-called "dark protein space". So far, only four *de novo *protein structures have been experimentally approximated. Low homology, presumed high disorder and limited structures result in low confidence structural predictions for *de novo* proteins in most cases. Here, we look at the most widely used structure and disorder predictors and assess their applicability for *de novo *emerged proteins. Since AlphaFold2 is based on the generation of multiple sequence alignments and was trained on solved structures of largely conserved and globular proteins, its performance on *de novo *proteins remains unknown. More recently, natural language models of proteins have been used for alignment-free structure predictions, potentially making them more suitable for *de novo* proteins than AlphaFold2.

**Methods: **We applied different disorder predictors (IUPred3 short/long, flDPnn) and structure predictors, AlphaFold2 on the one hand and language-based models (Omegafold, ESMfold, RGN2) on the other hand, to four de novo proteins with experimental evidence on structure. We compared the resulting predictions between the different predictors as well as to the existing experimental evidence.

**Results: **Results from IUPred, the most widely used disorder predictor, depend heavily on the choice of parameters and differ significantly from flDPnn which has been found to outperform most other predictors in a comparative assessment study recently. Similarly, different structure predictors yielded varying results and confidence scores for *de novo* proteins.

**Conclusions: **We suggest that, while in some cases protein language model based approaches might be more accurate than AlphaFold2, the structure prediction of *de novo* emerged proteins remains a difficult task for any predictor, be it disorder or structure.

## Introduction

The existence of proteins arising from non-coding parts of the genome, also known as *de novo* emergence, was once considered almost impossible [ Zuckerkandl, 1975, Jacob, 1977]. However, with the completion of the yeast genome project [ Dujon, 1996] and the discovery of “orphans”, which are defined as proteins lacking any detectable homology to proteins in sister species, the concept of *de novo* protein emergence called for a reevaluation. Among orphan proteins, *de novo* proteins are unique, as they can be shown using further methods including synteny analysis, to have been born from formerly non-coding DNA [ Vakirlis et al., 2020]. Accordingly, their sequence composition might resemble proteins with random sequences, yet to an unknown degree [ Oss and Carvunis, 2019, Bornberg-Bauer et al., 2021]. In particular, in several studies *de novo* proteins have been predicted to be highly disordered which can at least partially be attributed to their high GC (guanine-cytosine) content [ Wilson et al., 2017, Landry et al., 2015, Basile et al., 2017]. Other studies reported *de novo* proteins to contain a lower proportion of structural disorder [ Xie et al., 2019] than conserved proteins, while yet other studies report no significant difference in predicted disorder between *de novo* and conserved proteins [ Schmitz et al., 2018, Dowling et al., 2020]. The differences between these findings may be caused by the usage of different species and age groups studied in the datasets, but also to different methods used.

### Structure predictors and *de novo* proteins

Experimental structure determination of *de novo* proteins is still in its infancy, due to difficulties in purification and methodological limitations [ Eicholt et al., 2022]. Therefore, several studies include computational structure [ Vakirlis et al., 2020; Lange et al., 2021] and disorder predictions [ Schmitz et al., 2018, Xie et al., 2019]. However, most structure predictors are based on multiple sequence alignments (MSA) and training sets containing only structures of conserved proteins [ Jumper et al., 2021, Erdős et al., 2021, Hu et al., 2021]. While these methods certainly provide information on a wide array of protein properties, it is not yet clear how reliable these methods are for proteins with no detectable homology to known proteins, such as *de novo* proteins, but also random sequence proteins or *de novo* designed proteins [ AlQuraishi, 2021]. A more reliable option for predicting *de novo* protein structures could be programs based on protein language models (pLM) since these models do not require any MSA [ Michaud et al., 2022]. Instead, pLMs have learned general sequence architectures in proteins and how these relate to structures or structural elements. Using the language analogy, this is similar to learning grammar and building whole sentences from single words and words from letters [ Michaud et al., 2022, Chowdhury et al., 2022a, Wu et al., 2022a, Lin et al., 2022a]. While structural properties have been computationally analysed for several sets of *de novo* proteins [ Dowling et al., 2020, Heames et al., 2020, Wilson et al., 2017, Carvunis et al., 2012], only four *de novo* protein structures have been experimentally characterised [ Lange et al., 2021, Bungard et al., 2017, Matsuo et al., 2021; Her et al., 2019]. To date, no confirmed *de novo* protein structure has been completely solved experimentally.

### Structurally described *de novo* proteins

**AFGP** is a *de novo* emerged antifreeze glycoprotein family in Arctic codfish [ Baalsrud et al., 2018]. The family emerged *de novo* in the arctic codfish lineage around 15 mya (million years ago) with extant protein variants existing in several arctic codfish [ Baalsrud et al., 2018]. AFGP enables arctic codfish to survive the subzero temperatures of their biotope by preventing accumulation of ice crystals in the blood [ Cheng, 1998]. The secretion of AFGP into the blood is induced by a signal peptide, that is followed in the sequence by a post-translationally removed short glutamine-rich region, and T-(A/P)-A repeats up to 200 amino acids long [ Zhuang et al., 2019]. The Threonine residues of the repeats are glycolysated and bind to the surface of emerging ice crystals. AFGP blocks thereby the addition of water molecules to the ice crystal and decreases the freezing point of the blood serum. [ Cheng, 1998, Devries, 1971]. Nuclear magnetic resonance (NMR) spectroscopy of *de novo* emerged AFGP from *Boreogadus saida* revealed that AFGP is a highly dynamic and mostly disordered protein that can form polyproline II helices [ Her et al., 2019]. The AFGP in Antarctic notothenioid fish, while not emerged *de novo* but from a trypsinogen-like serine protease gene [ Cheng, 1998, Chen et al., 1997, Weisman, 2022], exhibits similar dynamic behaviour of the convergently emerged repetitive region [ Giubertoni et al., 2019].

**Bsc4** is a *de novo* gene specific to Baker’s yeast *Saccharomyces cerevisae* with a transcribed locus in homologous species, while lacking an open reading frame (ORF) in all transcripts except in *S. cerevisiae* [ Cai et al., 2008]. Protein expression of Bsc4 is upregulated during the stationary growth phase of Baker’s yeast. The deletion of Bsc4 is lethal when combined with the deletion of the conserved genes RPN4 and DUN1, but not contrariwise. RPN4 and DUN1 play both a role in DNA repair pathways [ Cai et al., 2008, Pan et al., 2006, Li et al., 2010]. This could indicate an important role of Bsc4 in the DNA repair pathway of yeast. The 132 amino acid long protein was analysed using tryptophan fluorescence and near-ultra violet (UV) circular dichroism (CD) and is considered to be of a molten globule structure consisting of abundant β -sheets while lacking tight packaging. According to ion mobility-mass spectrometry, Bsc4 can build homopolymer assemblies up to hexamers [ Bungard et al., 2017].

The role of the putative *de novo* protein **Goddard** was detected using fertility screens in *Drosophila melanogaster* [ Gubala et al., 2017]. Null-alleles of endogenous Goddard render male *D. melanogaster* infertile while not affecting viability. Using a combination of antibody staining and confocal microscopy, Goddard was found to localise to elongated sperm axonemes. The absence of Goddard results in failed individualisation of spermatids and therefore causes sterility in male fruit flies. The structure of Goddard was analysed using a combination of CD, thermal shift assay (TSA), NMR and *ab initio* structure prediction followed by molecular dynamics simulations [ Lange et al., 2021]. All methods indicate a central α -helix but high amounts of disorder in the rest of the Goddard protein. The central α -helix is conserved in Goddard orthologs and has been retained in the structure for at least 50 my, according to ancestral sequence reconstruction [ Lange et al., 2021].

The human specific *de novo* protein **NCYM** is the cis-antisense transcript of the MYCN oncogene. Both genic sequences are overlapping, but their coding regions do not overlap [ Weisman, 2022, Suenaga et al., 2020]. NCYM was the first *de novo* gene whose role in cancer progression was detected *in vivo* and has been structurally analysed [ Matsuo et al., 2021]. The SUMO-tagged NCYM protein was subjected to vacuum-UV CD and measurements were evaluated using an early neural network [ Matsuo et al., 2008]. The neural network subtracted the structural content of the SUMO-tag, thereby elegantly bypassing the cleavage of the tag from the 109 amino acid long NCYM [ Matsuo et al., 2021, 2008]. According to the predictions enhanced with CD data, NCYM is mostly disordered but contains several stretches of α -helices and some smaller β -sheets [ Matsuo et al., 2021].

### Relevance of disorder for *de novo* proteins

The structure-function paradigm suggests that a protein needs a defined structure to be functional [ Wright and Dyson, 1999]. However, research on disordered proteins demonstrated that this paradigm does not always hold up and that disordered proteins can carry out important biological functions too [ Uversky and Dunker, 2010]. For example, many binding motifs are located in disordered protein regions and disordered proteins are known to be involved in signalling pathways [ Ali et al., 2020]. However, it is widely asserted that a defined tertiary structure is complex and presumably difficult to attain from scratch, *i.e.,* without adaptation. Therefore, *de novo* proteins are often assumed to contain little structural content [ Bungard et al., 2017, Wilson et al., 2017, Schmitz et al., 2018]. Many *de novo* protein studies have included disorder predictions in their analyses [ Schmitz et al., 2018, Xie et al., 2019, Carvunis et al., 2012, Wilson et al., 2017]. During protein evolution, such a lack of well-defined structure might even be an advantage for newly emerging proteins under some circumstances. Indeed, highly disordered proteins were shown to be soluble and less prone to aggregation [ Linding et al., 2004], which has been described as a favoured trait in protein evolution. Since solubility is required for most protein functions a majority of protein sequences have evolved towards lower aggregation propensities [ Monti et al., 2021].

### Disorder prediction tools

The amount of disorder of a protein is relatively straightforward to predict from its amino acid sequence. Several algorithms are available as online interfaces or local programs [ Dosztányi et al., 2005, Erdős et al., 2021, Necci et al., 2021, Hu et al., 2021, Hanson et al., 2019]. IUPred is among the most frequently used disorder predictors, especially in *de novo* protein studies [ Schmitz et al., 2018, Wilson et al., 2017, Xie et al., 2019, Erdős et al., 2021]. IUPred is not based on evolutionary information but physical properties of the amino acids to be structure or disorder promoting, by using energy estimations of the single amino acids in the sequence [ Erdős et al., 2021, Dosztányi et al., 2005]. These energy estimations are derived from known contacts between amino acids in experimentally determined structures of globular proteins. This results in a 20x20 matrix containing energy estimations for each pair of amino acids. The final disorder probability for each residue depends on the energy estimation of the specific amino acid and its neighbouring residues. Accordingly, IUPred appears to be most suitable for proteins without known homologs.

Recently, the final results of Critical Assessment of protein Intrinsic Disorder prediction (CAID) [ Necci et al., 2021], demonstrated that there are many precise machine learning-based disorder predictors available that outperform IUPred in accuracy [ Hanson et al., 2019, Hu et al., 2021]. However, most of the top disorder predictors rely on evolutionary information, which may not be ideal for prediction of *de novo* proteins and other unusual sequences. flDPnn is among the few top disorder predictors that do not rely on evolutionary information, making it a promising predictor for *de novo* proteins. The true positive rate of predicted disorder is highest for flDPnn [ Hu et al., 2021] when compared to the other predictors (SPOT, IUPred “long” and “short”).

### Structure prediction with AlphaFold2

Structural biology has changed with the advent of DeepMind’s AlphaFold2 (AF2) [ Jumper et al., 2021] and structure predictors gained ground for many different research areas [ Lupas et al., 2021; Marx, 2022]. As of now, the AF2 protein structure database, a joint project of DeepMind and EMBL-EBI, contains more than 100 million high quality predicted protein structures, *e.g.* from *Homo sapiens* & *D. melangoaster* [ Varadi et al., 2021]. The abundant high-quality predictions in the AF2 PDB have already been leveraged for improved geometric pre-training of structure predictors of the next generation [ Zhang et al., 2022]. Until yet training was only limited to experimentally solved structures [ Zhang et al., 2022]. Novel structure predictors such as AF2 are particularly promising for studying *de novo* proteins due to the aforementioned lack of experimentally determined structures. However, AF2 has its own limitations. The properties of *de novo* proteins such as high disorder, short length and lack of homologous proteins make structure prediction of those *de novo* proteins a challenging task for AF2. Accordingly, results must be interpreted with caution [ Monzon et al., 2022]. The lack of homologous sequences in particular might pose a problem for AF2 since it is based on co-evolutionary data extracted from MSAs. AF2 uses correlations of co-occurences between amino acids in an MSA to deduce the proximity of those amino acids in the protein structure [ Jumper et al., 2021; Michaud et al., 2022]. *De novo* proteins do not necessarily lack homology entirely, since they can also appear in a whole lineage, as in the case for AFGP. In those cases, an MSA could provide co-evolutionary data to predict secondary structure elements but likely not for the abundant disordered regions in which the assumption of positional homology could be violated [ Lindorff-Larsen and Kragelund, 2021]. Disordered regions are highly flexible in space, while predictions based on MSAs assume that the amino acid position in a sequence correlates to a fixed position in the structure [ Lindorff-Larsen and Kragelund, 2021]. Nevertheless, *de novo* proteins are assumed to be mutationally remote in sequence space (and therefore evolutionary unrelated) to areas of well characterised protein families in structural space. Therefore, recent structure prediction programs based on protein language models (pLMs) could yield more realistic results for *de novo* protein structure since they are alignment-free.

### Predictions of *de novo* proteins

We will summarise existing structural evidence for different *de novo* proteins and methodological limitations, with a focus on the most widely used disorder and structure predictors, IUPred3 and AlphaFold2, respectively. A major caveat for disorder comparison is that computational predictions of *de novo* protein properties are difficult to compare between studies, because of differences in parameters used [ Schmitz et al., 2018]. Here, we used four experimentally characterised, or rather approximated, *de novo* proteins to illustrate that results of different prediction algorithms differ significantly in most cases, and do not always align well with the experimental evidence at hand. Finally, we will focus on longer standing questions on structure predictions of *de novo* proteins and on novel questions that were raised with the advancement of machine learning (ML) based structure predictions: Specifically we ask, how reliable structure predictions for *de novo* proteins are and what possible pitfalls during analysis of those predictions may arise. Enabled by the advancement of the structure prediction field, the structural analysis of *de novo* proteins will thus bring more light into the “dark protein space”, the hitherto non-characterised region of sequence space. Therefore, novel structures and folds could be discovered and provide new starting points for protein engineering and deeper insights on protein evolution.

## Methods

### Protein sequences

Only *de novo* protein sequences with experimental evidence on structure were taken for analyses. For this purpose, available peer-reviewed publications on *de novo* emerged proteins were manually screened for candidates [ Weisman, 2022, Bornberg-Bauer et al., 2021]. After screening literature for appropriate candidates and removing i) *de novo* proteins without structural information and ii) falsely identified *de novo* emerged candidates, the *de novo* protein sequences were downloaded from UniProt (RRID:SCR_002380), accessed in December 2022. The UniProt accession numbers can be found in Table 1 and all sequences used are included in the underlying data as fasta files. Eight conserved proteins with experimentally determined structures containing different amounts of disorder, four with low and four with relatively high amounts of disorder were taken as controls. The observed values for the fraction of residues in disordered regions were taken from MobiDB (RRID:SCR_014542). For accession numbers and species of origin, see Table 1. Amino acids were counted with a custom Python 3.10 (RRID:SCR_008394) script available on Zenodo: https://doi.org/10.5281/zenodo.7615407 and zivgitlab/l_eich04/structure_predictions_de_novo.

**Table 1.** Proteins predicted.

Protein	Species	UniProt	amino acids
AFGP	*Boreogadus saida* (Polar cod)	A0A481T066	701 aa
Bsc4	*Saccharomyces cerevisiae* (Baker’s yeast)	P53841	131 aa
Goddard	*Drosophila melanogaster* (Fruit fly)	Q9VUG4	113 aa
NCYM	*Homo sapiens* (Human)	P40205	109 aa
AFGP polyprotein	*Dissostichus mawsoni* (Antarctic cod)	O13083	722 aa
Antifreeze glycopeptide	*D. mawsoni* (Antarctic cod)	Q90401	72 aa
AF9	*H. sapiens* (Human)	P42568	568 aa
Nop10	*S. cerevisiae* (Baker’s yeast)	Q6Q547	58 aa
Alphasynuclein	*H. sapiens* (Human)	P37840	140 aa
Cellular tumor antigen p53	*H. sapiens* (Human)	P04637	393 aa
Cytochrome P450	*Sulfurisphaera tokodaii*	Q972I2	367 aa
Bifunctional epoxide hydrolase	*H. sapiens* (Human)	P34913	555 aa
Interferon gamma	*Paralichthys olivaceus* (Bastard halibut)	B3IXK1	198 aa
Myoglobin	*Physeter macrocephalus* (Sperm whale)	P02185	154 aa

### Disorder predictions

Disorder predictions were performed locally using IUPred3 [ Erdős et al., 2021] (RRID:SCR_014632), using the parameters “short” and “long” predictions and flDPnn [ Hu et al., 2021] using default parameters. The fraction of residues in a disordered region (referred to simply as fraction from hereon) was determined by calculating the average of the binary predictions for disorder in flDPnn. For IUPred, the binary predictions were calculated first by assigning the value 1 if predicted disorder was >0.5 , and 0 if predicted disorder was <0.5 and then averaged to get the fraction of residues in disordered regions. Statistical analysis and plots were done in RStudio 4.2.2. (RRID:SCR_000432) [ RStudio Team, 2020, R Core Team, 2022]. To determine whether the observed differences were significant (p-value < 0.05), the Kruskal-Wallis rank sum test followed by the Dunn test were performed and p-values adjusted using Holm method from FSA package [ Ogle et al., 2022]. Plots were generated using the ggplot2 package [ Wickham, 2016]. The code used in R is available as “R_stats_plots.txt” on Zenodo: https://doi.org/10.5281/zenodo.7615407 and on zivgitlab/l_eich04/structure_predictions_de_novo. All software tools used are freely available.

### Structure predictions

Structural predictions were performed using AlphaFold v2.1.1 on High Performance Computing Cluster PALMA II (University of Muenster). RGN2 (Number of recycles 1), OmegaFold (Number of cycles 4) and ESMfold (Number of cycles 3) predictions were performed using respective Google Colabs (RRID:SCR_018009) [ Chowdhury et al., 2022b, Wu et al., 2022b, Lin et al., 2022b]. For each the standard number of cycles/recycles were chosen. Predictions with the highest mean pLDDT were selected. The pLDDT of different segments were examined with ChimeraX 1.5 [ Pettersen et al., 2021] (RRID:SCR_015872) and the command’color bfactor palette alphafold’. PyMOL 2.5.2. [ Schrödinger, LLC, 2015] (RRID:SCR_000305) was used for structural alignments and visualizations. AlphaPickle [ Arnold, 2021] was used to pull pLDDT values for each residue from the b factor column of PDB files. Two N-terminal residues were removed for predictions of AlphaFold2, Omegafold and ESMfold since RGN2 predictions exclude the last two N-terminal residues [ Floristean, 2022]. Violin plots were created using Python 3.10 (RRID:SCR_008394) with libraries matplotlib [ Hunter, 2007] (RRID:SCR_008624) and pandas [Wes McKinney, 2010] (RRID:SCR_018214). Kruskal-Wallis rank sum test and Dunn test were performed and p-values adjusted using Holm method in RStudio [ RStudio Team, 2020] as described for the disorder predictions. AlphaFold2 predictions of AFGP polyprotein (O13083) and Antifreeze glycopeptide (Q90401) of *Dissostichus mawsoni* (Antarctic cod) were not performed but downloaded from AlphaFold Protein Structure Database.

All software tools used are freely available. All code and original result files are available in the extended data on Zenodo: https://doi.org/10.5281/zenodo.7615407. Code is additionally available on zivgitlab: https://zivgitlab.uni-muenster.de/l_eich04/structure_predictions_de_novo.

## Results

### Comparing disorder predictions of *de novo* proteins

**Figure 1.**
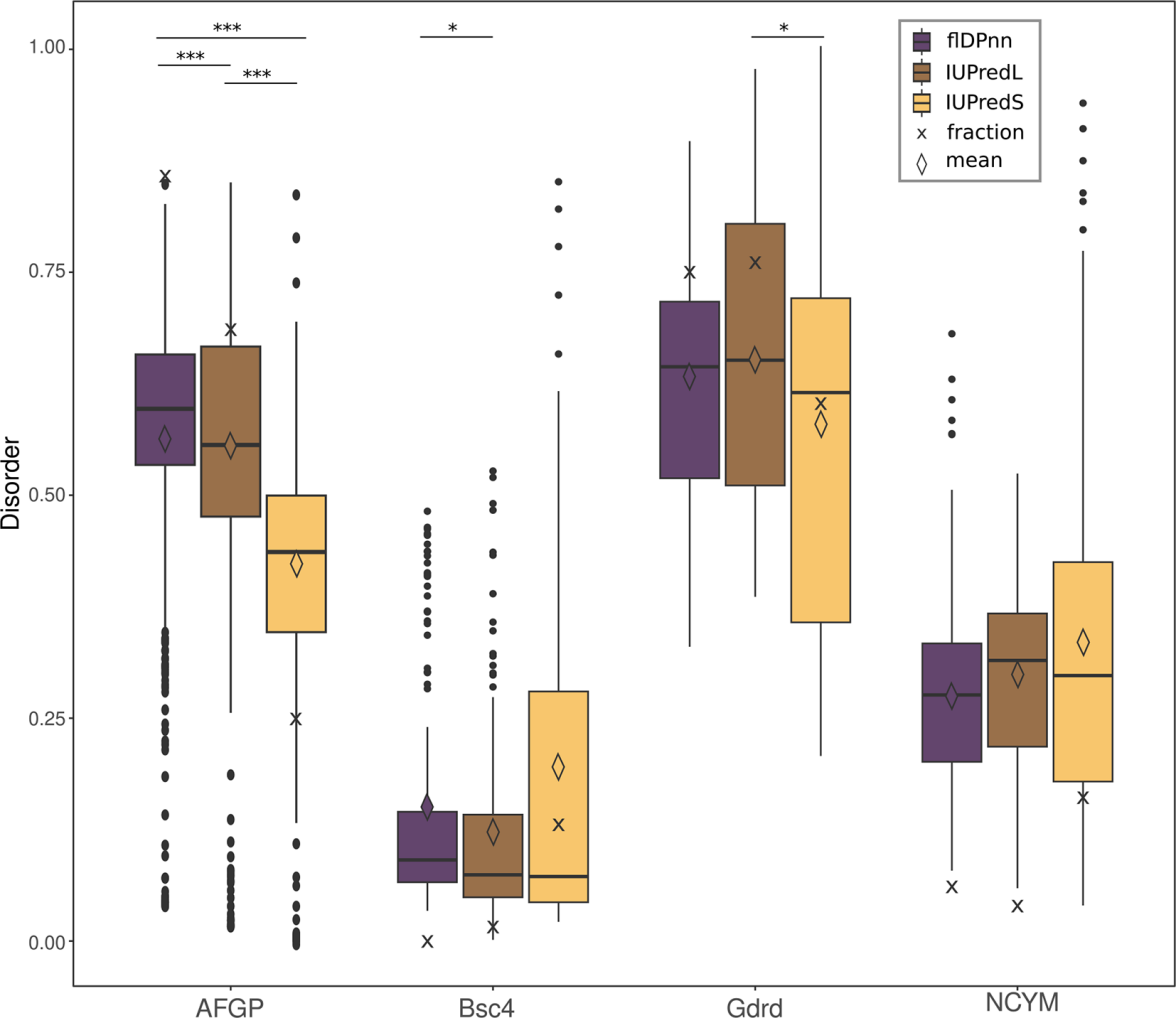
Comparison of different disorder predictions for *de novo* proteins: The predictors flDPnn and IUPred long (L) and short (S) have been used for disorder predictions of the four *de novo* genes AFGP, Bsc4, Goddard (Gdrd) and NCYM. Mean values are displayed as diamond shapes, median as lines and crosses display the fraction of disordered residues. Significant differences between the disorder predictors are indicated by stars (***<0.0005; **<0.005; *<0.05).

Here, we compare the performance of flDPnn [ Hu et al., 2021], which performs best according to CAID, to the latest version of IUPred [ Erdős et al., 2021], the most widely used predictor. We focus on *de novo* proteins that were experimentally characterised, namely AFGP, Bsc4, Goddard and NCYM (see Figure 1). According to experimental evidence, all four *de novo* proteins contain disordered regions. When predicting the disorder of AFGP with flDPnn as described in the methods, around 80 % of residues are predicted to be disordered. IUPred “long” (IUPredL) predicts around 70 % and IUPred “short” (IUPredS) only 25 %. Here, the biggest and most significant difference can be observed between the predictors, with all p-values < 0.005. This can be partly attributed to AFGP being by far the longest protein used in the analyses with 700 amino acids (see Table 1). **Bsc4** predictions are highly similar between the three predictors and indicate low amounts of disorder. The median value is around 0.1 for all predictors while the fraction and mean show more variation from 0-13 % and 0.12-0.19 respectively. All predictors that were used on the Goddard sequence here result in high disorder with around 75 % of all residues in a disordered region and a mean score of about 0.7 for Goddard predictions. Predictions of Goddard differ significantly between IUPredS and IUPredL (p-value = 0.0157), highlighting the importance of the right choice of parameters. **NCYM** disorder predictions recognise 10 % of residues as disordered with mean probability for disorder of around 0.25. There is no significant difference between the disorder predictors, which can be partially attributed to the short length of the protein (109 amino acids).

Probabilities for disorder in all *de novo* protein sequences, except NCYM, differ significantly between the predictors with p-values below 0.05 as shown in Figure 1 **and underlying data**. Most importantly, the fraction of disordered residues varies greatly depending on the predictor that was used here.

As shown in Figure 1 the difference between fraction of disordered residues and mean probability for disorder over all residues in the sequence can deviate significantly. While both values are indicators for disorder in a protein, they have slightly different implications. On the one hand, when only the average probability for disorder of all residues is reported, little information on actual amount of disorder in a protein is gained. It is impossible to distinguish between a theoretical protein with some highly disordered (probability close to 1) and some highly structured regions (probability close to 0) and a protein with ambiguous probability for disorder (probability around 0.5) in the whole sequence. Looking at the predictions performed with the *de novo* proteins, flDPnn predicts very similar average probabilities for disorder in AFGP and Goddard (0.57 and 0.61). The fraction of residues in a disordered region not only differs more between the two than the average disorder, but the trend is actually reversed (82 and 75 %). Goddard is predicted to contain 75 % of disordered residues and AFGP 82 %. On the other hand, when using the fraction of residues that are predicted to be disordered in the protein, the minor differences in probabilities for disorder disappear. For example, a theoretical protein that is just below the threshold for a disordered region of 0.5 in the majority of the sequence, is indistinguishable from a protein with a probability for disorder of 0 across the whole sequence. The *de novo* proteins NCYM and Bsc4 predicted here have a very similar fraction of disordered residues, but judging from the average probability they seem to differ much more.

**Figure 2.**
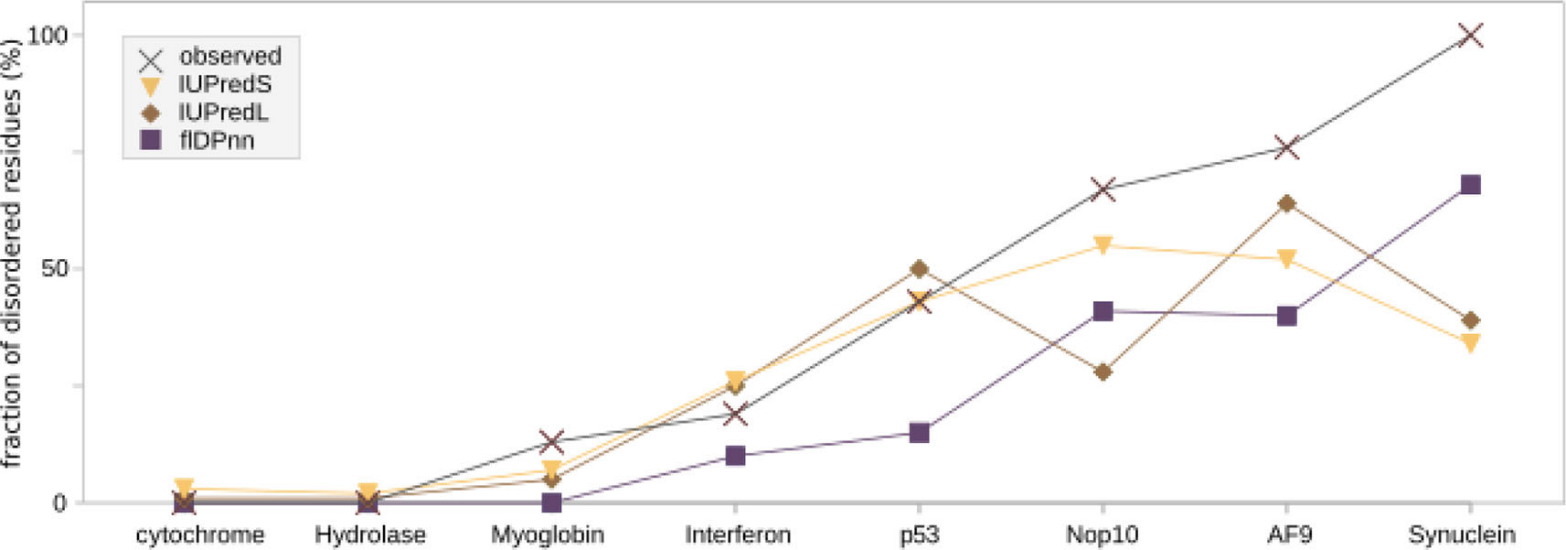
Predicted percentage of residues in a disordered region for experimentally solved protein structures as controls. The disorder for four highly structured and four highly disordered proteins with experimentally resolved structures was predicted with IUPredS, IUPredL and flDPnn. The proteins are ordered from low disorder on the left to high disorder on the right. While flDPnn predicts the disorder for all lower than observed values, the trend remains the same. IUPredL and IUPredS are closer to the observed values for structured proteins, but deviate from the trend for disordered proteins.

For a more general comparison, we took eight conserved and experimentally solved structures and applied the same prediction algorithms. All eight proteins have varying amounts of disorder based on the PDB structures which we collected from the disorder database MobiDB. The observed disorder is indicated in Figure 2 and is compared to the values predicted by the three programs that were also applied to the *de novo* proteins before (IUPredL, IUPredS and flDPnn). Four of the proteins contain low amounts of disorder below 25 % according to the experimentally determined structures. Results of all three disorder predictors are close to the observed values. The four proteins that contain higher amounts of disorder (p53, Nop10, AF9 and alpha-synuclein), vary much more between the predictors and have higher amounts of observed disorder than is predicted.

### Structures and pLDDT of *de novo* protein predictions

Here, we will present the structure predictions of *de novo* proteins AFGP from *B. saida*, Bsc4, Goddard and NCYM [ Her et al., 2019, Bungard et al., 2017, Lange et al., 2021, Matsuo et al., 2021] with AF2, OF, RGN2 and ESMFold [ Jumper et al., 2021, Wu et al., 2022a, Chowdhury et al., 2022a, Lin et al., 2022a]. As mentioned before, there is no experimentally determined structure of a *de novo* evolved protein that can serve as ground truth when comparing prediction programs. All programs provide a predicted local distance difference test (pLDDT) [ Jumper et al., 2021, Mariani et al., 2013] based on the AF2 structure module to evaluate the prediction confidence of each residue of the model.

It is important to note here that pLDDT is a confidence measure of each program for the predictions performed by itself and not to compare the confidence of predictions of different programs to each other. In the following, when we compare the pLDDTs of different programs we are thereby not assessing which program provides the most reliable prediction. Also, low pLDDT can be an indicator of high disorder [ Akdel et al., 2022, Ruff and Pappu, 2021].

Additionally as controls, we performed structure predictions in the same manner as for the *de novo* proteins, for evolutionary conserved and both structurally solved and experimentally confirmed intrinsically disordered proteins (IDPs); p53, Nop10, AF-9 and Alpha-synuclein ( Figure 4 & Table 1).

**Figure 3.**
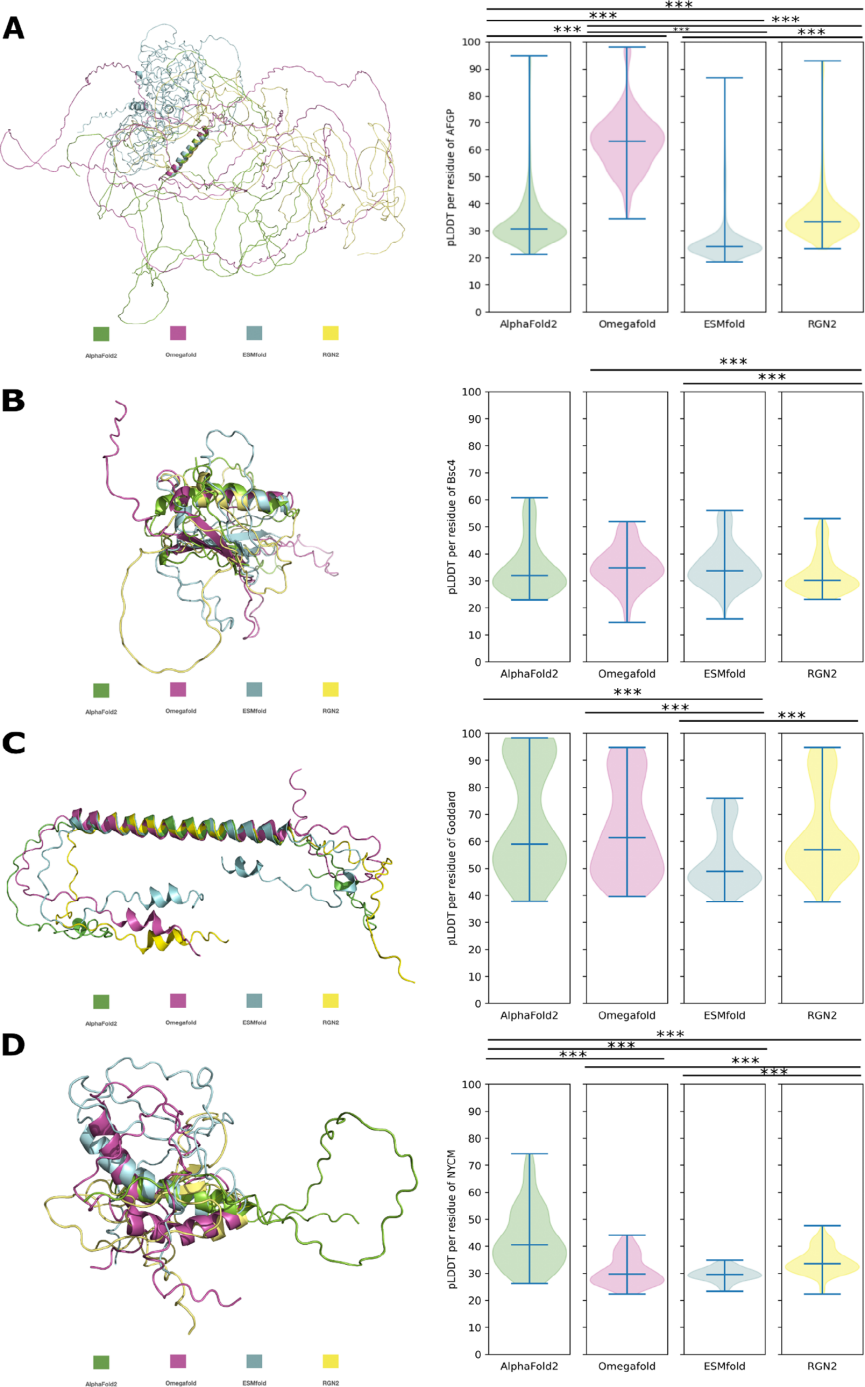
Comparison of different structure predictions for *de novo* proteins: Structural alignment of all models to a respective secondary element of the AF2 prediction and violin plots of pLDDT per residue of each model. A: AFGP, to α -helix (M1-A28), C α -RMSD = 0.663Å. B: Bsc4, to α -helix (K62-R83), C α -RMSD = 1.603Å. C: Goddard, to α -helix (S38-I80), C α -RMSD = 0.770Å. D: NCYM, to α -helix (N50-E66), C α -RMSD = 5.278Å. Significant difference in pLDDT is indicated by *** (p-value < 0.0005).

The pLDDT values for the predictions of **AFGP** from *B. saida* ( Figure 3A) are significantly different from each other’s prediction. We found that all programs predict an N-terminal α -helix while the rest of the structure is ribbon-like, indicative of disorder. Only ESMfold predicts three additional shorter helices (T222-T225, T414-A418, T672-A680). The predictions of AFGP show differing pLDDT between predictors while all predictions effectively display high levels of disorder. The pLDDT values of **Bsc4** are more similar to each other except for predictions obtained from OF and ESMfold. These two differ significantly in pLDDT from those obtained with RGN2 and different secondary elements are predicted ( Figure 3B). AF2 predicts smaller β -sheets and RGN2 does not predict any β -sheets. However, in a lower pLDDT-ranked AF2 structure (see extended data), the determined β -sheets are similar predicted and almost identical to those by OF and ESMfold. Predictions of Bsc4 have similar pLDDT values while the underlying predicted structures are not. **Goddard** is structurally composed of a confirmed central α -helix and disordered termini [ Lange et al., 2021]. The predicted structures of Goddard are similar by eye ( Figure 3C) and if the models are structurally aligned to the central α -helix of the AF2 model as a target this similarity becomes even more apparent ( Figure 3C, C α -RMSD = 0.770Å). AF2, OF and RGN2 predict the majority of the helix with very high confidence which decreases only towards the termini when employing RGN2 and OF. ESMfold predicts the structure of Goddard only with low to very low confidence, with significant difference in pLDDT to other predictors.

For **NCYM** ( Figure 3D) all programs except OF predict one α -helix, while OF predicts two. The α -helix predicted by OF and AF2 is longer than the one predicted by RGN2. OF predicts an additional α -helix. RGN2 is the only method that predicts β -sheets (R60-C64, C104-I107). These β -sheets overlap with the α -helices predicted by the other programs, explaining the lower RMSD (C α -RMSD = 5.278Å). In this case, the pLDDT of AF2 is higher than the pLDDT of the other programs, which have comparably low values for predictions of NCYM.

**Figure 4.**
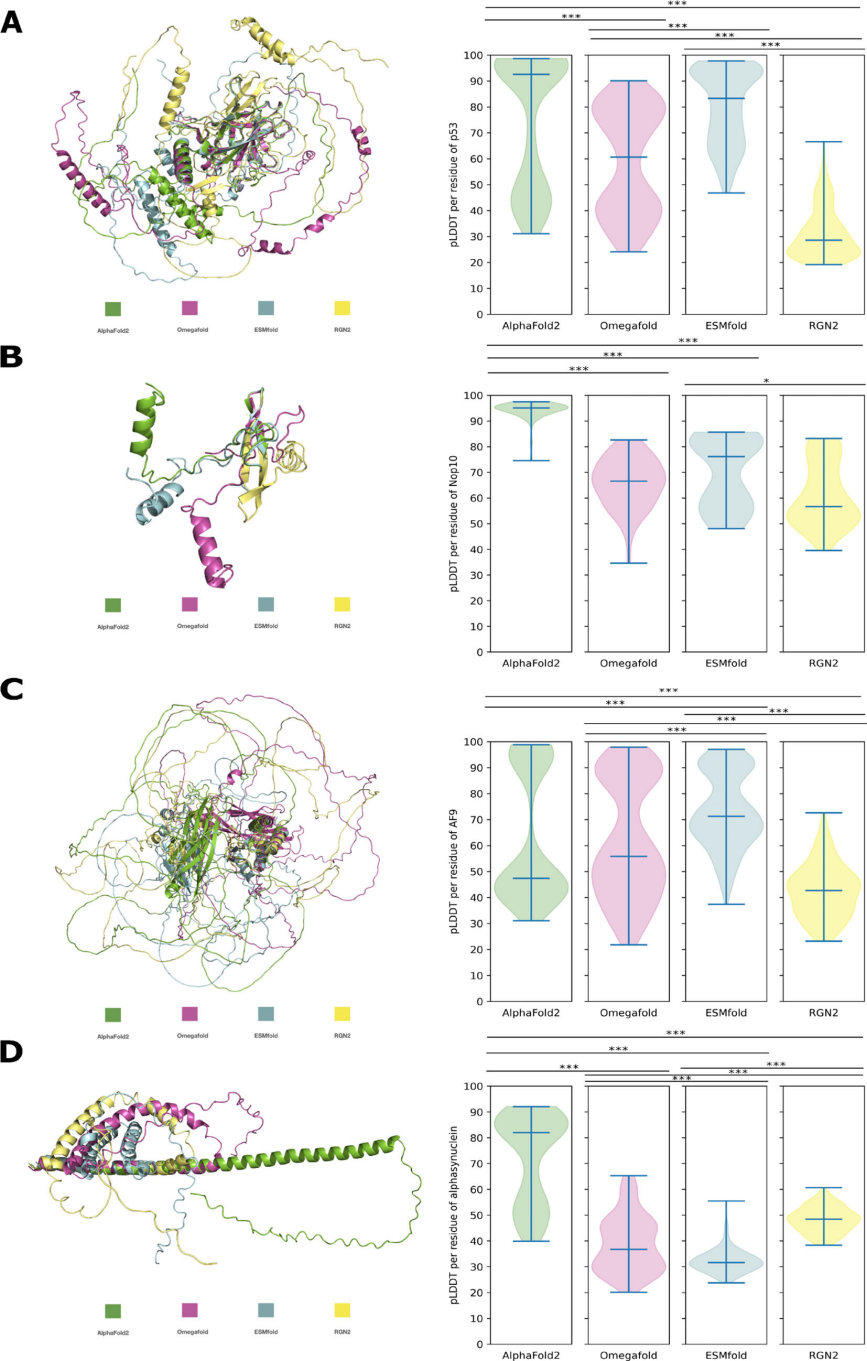
Comparison of pLDDT of different structure predictions for experimentally confirmed disordered proteins: Structural alignment of all models to a respective secondary element of the AF2 prediction and violin plots of pLDDT per residue of each model. A: p53, to β -sheets (C123-E203), C α -RMSD = 19.198Å. B: Nop10, to β -sheets (M4-T16), C α -RMSD = 0.269Å. C: AF9, to to α -helices (K500-S566), C α -RMSD = 1.558Å. D: alphasynuclein, to α -helix (M1-G40), C α -RMSD = 1.140Å. Significant difference in pLDDT is indicated by *, **, *** (p-value < 0.05 < 0.005 < 0.0005).

The question remained, if both the overall results of structure predictions of *de novo* proteins and their variations are a consequence of i) disorder level and/or of ii) lack of sequence homology. Therefore, we performed structure predictions of p53, Nop10, AF9 and alpha-synuclein with the same tools as before (AF2, OF, ESMfold, RGN2). These control proteins are all evolutionary conserved and are experimentally confirmed intrinsically disordered proteins (IDPs). For each of the four control IDPs the predicted secondary structure elements are approximately in the same position for all prediction tools. Only lengths of secondary elements and of ribbon-like structures, indicating disorder, are variying between each prediction ( Figure 4). The respective predictions of all four control IDPs show broadly significant differences in pLDDT but not in all cases ( Figure 4). The number of significant differences in pLDDT for predictions of IDPs is lower than for *de novo* protein predictions ( Figures 3 and 4). In general the pLDDT values for the predictions of experimentally conserved IDPs are higher than for *de novo* proteins ( Table 2).

**Table 2.** mean pLDDT for each structure predictor of each protein.

Protein	AF2	OF	ESMfold	RGN2	mean pLDDT (protein)
AFGP	30.67	62.99	24.04	33.29	32.75
Bsc4	32.08	34.74	33.68	30.21	32.78
Goddard	58.97	61.45	48.85	57	56.235
NCYM	40.59	29.83	29.6	33.62	31.815
**mean pLDDT per program ( *de novo*)**	36.335	48.095	33.65	32.765	
Nop10	95.1	66.575	76.135	56.72	73.78
AF9	47.445	55.85	71.27	42.7	53.48
alphasynuclein	82.06	36.76	31.65	48.4	45.095
p53	92.61	60.65	83.33	28.59	61.245
**mean pLDDT per program (IDPs)**	87.335	63.6125	73.7025	45.55	
**mean pLDDT per program (all)**	53.2075	61.05	42.805	37.74	

## Discussion

### Disorder in *de novo* proteins

Research on functional disordered proteins is increasing and so is the need to structurally characterise and detect disordered protein regions [ Alderson et al., 2022, Lindorff-Larsen and Kragelund, 2021, Ali et al., 2020, Bruley et al., 2022]. For newly detected but also newly emerged proteins, as *de novo* proteins often are, disorder is an interesting hallmark to investigate because disorder promotes high solubility, disfavours aggregation [ Linding et al., 2004, Monti et al., 2021], and at the same time, is often associated with a high density of binding motifs which make the protein amenable to many regulatory processes [ Ali et al., 2020]. Disorder predictions are therefore often used to gather information about *de novo* proteins by comparing them to: i) conserved proteins [ Xie et al., 2019, Carvunis et al., 2012], ii) different age groups in *de novo* proteins [ Schmitz et al., 2018, Wilson et al., 2017, Carvunis et al., 2012, Dowling et al., 2020] and iii) random sequence proteins [ Heames et al., 2022]. With many studies relying on the disorder predictions of *de novo* proteins and only few attempts to experimentally characterise their disorder [ Heames et al., 2022, Bungard et al., 2017], it is paramount that the predictors used are precise enough for *de novo* protein sequences to allow for the conclusions drawn. Further, to compare predictions not just in single studies but more easily between different studies, consensus about prediction methods and parameters is needed.

### Comparing disorder predictions with experimental evidence

Comparing disorder predictions of the four *de novo* proteins with each other, the overall trend in all predictors and according to experimental data is the same. According to literature on experimental analyses of the *de novo* proteins [ Lange et al., 2021, Bungard et al., 2017, Matsuo et al., 2021, Her et al., 2019], the *de novo* proteins can be ordered by estimated amount of disorder to verify comparability of the different predictors. Bsc4 contains the least disordered residues, followed by NCYM with around half of the residues in disordered regions. Goddard is highly disordered, containing only one (long) helix, while AFGP has the highest amount of disorder among the here discussed *de novo* proteins. In most computational *de novo* protein studies, either the mean or the fraction are reported to use as a comparison between different classes of *de novo* proteins [ Xie et al., 2019, Schmitz et al., 2018, Dowling et al., 2020, Wilson et al., 2017]. When comparing these single disorder values for the *de novo* proteins at hand, only results from the fraction of residues in a disordered region predicted by flDPnn correspond to the experimental data. Overall, flDPnn slightly outperforms IUPred when comparing the disorder predictions with the experimentally characterised structures of *de novo* proteins. The same was observed in CAID [ Necci et al., 2021] where disorder predictions are assessed based on recently determined structures containing disordered regions. Equally, flDPnn predicted the right order from low to high disorder in the control proteins while IUPredS and IUPredL did not (see extended materials Figure 2). However, all three predictors resulted in lower disorder values for the highly disordered proteins than indicated by experimental data. The control proteins Nop10, AF9 and synuclein are mostly disordered proteins with over 67 % to 100 % of residues in disordered regions. All three predictors results in lower percentage of disordered regions predicted ranging from 28 % (IUPredL for Nop10) to 68 % (flDPnn for synuclein). The predictions of the control proteins with both homologous sequences as well as experimentally determined structures available, are close to the experimentally observed disorder for the more structured proteins. For cytochrome and the hydrolase all three predictors resulted in percentages of disorder close to zero in accordance with experimentally determined structures (see Figure 2). Predictions of the control proteins with high disorder were lower than observed experimentally, but nevertheless the order of proteins from low to high disorder between observed and flDPnn was the same. This indicates that not only the orphan status of *de novo* proteins pose a problem for disorder predictors. Also, the high amount of disorder that is a commonly associated trait in *de novo* proteins may be one of the hurdles in disorder prediction of *de novo* and orphan proteins. Therefore, for the prediction of protein disorder in orphan proteins, such as *de novo* proteins, or other proteins without homologous sequences available, like random sequence proteins or designed proteins, still more suitable predictors are needed. In the absence of such more applicable predictors, it seems advisable to obtain and provide, wherever possible, additional experimental evidence on structure.

### Use of parameters in disorder predictions

Predicted disorder for *de novo* proteins by IUPred, the most widely used program, differs significantly i) between results when used “short” vs. “long” prediction parameter and ii) to results from flDPnn, which is among the best disorder predictors according to CAID [ Necci et al., 2021, Hu et al., 2021]). While most studies on *de novo* proteins use IUPred, the use of “long” and “short” prediction varies from study to study, as well as the type of value (mean/median probability or fraction of disordered residues) that is eventually reported for comparison [ Schmitz et al., 2018, Wilson et al., 2017]. This poses another problem of comparability between different studies on *de novo* proteins. While most studies on *de novo* proteins use IUPred, there seems to be a disagreement whether the “long” or “short” parameter is most suitable. According to the authors of IUPred [ Dosztányi et al., 2005, Erdős et al., 2021], “short" disorder is used for small patches of disorder, for example in partially solved X-ray structures and generally predicts higher disorder at the N- and C-termini. Therefore, the same residues are predicted differently when placed at the termini of a sequence, rather than towards the centre. The “long” option should be used for global disorder in a protein. Accordingly, the “long” parameter prediction seems best suitable for predicting disorder if IUPred is deployed to *de novo* proteins. However, most studies favour the “short” prediction [ Lange et al., 2021, Bungard et al., 2017, Schmitz et al., 2018, Dowling et al., 2020] over the “long” prediction [ Xie et al., 2019]. Only few studies use both [ Basile et al., 2017], while others do not state explicitly which one was used [ Baalsrud et al., 2018, Wilson et al., 2017]. In these cases it must be assumed that the default “long” was applied.

Like other disorder and predictors, IUPred’s output assigns a probability for an amino acid being in a disordered region. A protein sequence of 100 amino acids accordingly results in 100 single probabilities for disorder. For easier comparison between multiple proteins, most studies [ Schmitz et al., 2018, Wilson et al., 2017, Xie et al., 2019] only report a single value per protein sequence, instead of the probabilities per residue. This reported value can either be the fraction of residues predicted to be in a disordered region [ Schmitz et al., 2018, Dowling et al., 2020, Basile et al., 2017], or the average or median value of probability for disorder over the whole sequence [ Wilson et al., 2017, Xie et al., 2019, Baalsrud et al., 2018]. The reported values of predicted disorder per protein reported within a study usually do not affect the analyses [ Schmitz et al., 2018]. However, the combination between different parameters chosen and different values reported for the disorder per protein makes it difficult to accurately compare results of disorder predictions between studies.

Therefore, both values (fraction and mean) should be reported when comparing disorder values between proteins as done for example in Eicholt *et al.* (2022). Similarly, when analysing single proteins, it is recommended to use multiple disorder predictors as implied for example in the MPI toolkit [ Alva et al., 2016]. For bulk comparisons between different sets of proteins this is not always possible. Therefore, the disorder algorithm should be chosen carefully with provision of insights from the most recent structure prediction assessment [ Necci et al., 2021]. However, all compared predictors here failed to predict disorder accurately compared to experimental evidence. The predictions of the control proteins with both homologous sequences as well as experimentally determined structures available, are close to the experimentally observed disorder for the more structured proteins. Predictions of the control proteins with high disorder were lower than observed experimentally, but nevertheless the order of proteins from low to high disorder between observed and flDPnn was the same. This indicates that not only the orphan status of *de novo* proteins pose a problem for disorder predictors. Also, the high amount of disorder that is a commonly associated trait in *de novo* proteins may be one of the hurdles in disorder prediction of *de novo* and orphan proteins. Therefore, for the prediction of protein disorder in orphan proteins, such as *de novo* proteins, or other proteins without homologous sequences available, like random sequence proteins or designed proteins, still more suitable predictors are needed. In the absence of such more applicable predictors, it seems advisable to obtain and provide, wherever possible, additional experimental evidence on structure.

### Comparing structure prediction programs

AF2 relies on an MSA to detect co-evolutionary patterns which is utilised to indicate the proximity of residues in space. More recently, protein language models, so called pLMs, have been employed for protein structure predictions. Structure predictors based on pLMs, such as Omegafold (OF) [ Wu et al., 2022a], ESMfold [ Lin et al., 2022a] and RGN2 [ Chowdhury et al., 2022a] are trained to fill in the blanks of masked sequences, thereby learning interconnections between residues in protein sequences [ Michaud et al., 2022]. This training is analogous to gap-filling exercises when learning a new language [ Ferruz and Höcker, 2022, Ofer et al., 2021]. OF, ESMfold and RGN2 combine their language models with the structure module of AF2. In their original publications, the three pLM predictors were also tested on a set of orphans and compared to the performance of AF2 on the same set. Nevertheless, in all studies a different depth of search was employed to classify sequences as orphans. For OF, recent additions to the PDB without homologs were selected. For ESMfold also recent additions to the PDB were selected, clustered with mmseqs (70% identity threshold) and HHblits was used to confirm zero hits. Additionally, AF2’s MSA generation on UniRef, MGnify and BFD was used to find sequences with <1,<10,<100 hits, and TMscore<0.5 for any structural template. TM score is a metric used to assess the similarity of two protein structures encoded by the same sequence, while TMscore=1 indicates identical structures [ Zhang and Skolnick, 2004]. For RGN2, orphans were defined as sequences with MSAdepth=1 across UniRef30, PDB70 and MGnify, meaning the resulting MSA consists only of the query sequence but no other [ Chowdhury et al., 2022a]. Only OF and RGN2 were tested on orphans with experimentally determined structures and predictions compared to those of AF2. While OF outperformed AF2 significantly in that OF predictions had higher TM-scores when compared to experimentally solved structures, RGN2 surpassed AF2 only slightly, which might be due to several reasons. First, OF’s ability to predict orphans was tested on recently solved structures that were neither part of the OF training set nor of the AF2 training set, while in the case for RGN2 the majority of orphans were in the training for both [ Ahdritz et al., 2022, Chowdhury et al., 2022a]. Second, the definition of orphans as consisting of an MSAdepth=1 in RGN2 might cause a bias towards short proteins and AF2 might be able to solve the global search problem for the energy minima of these short proteins despite AF2 not being optimised for short *de novo* proteins [ Eicholt et al., 2022]. While RGN2 and OF are based on different pLMs (AminoBERT [ Chowdhury et al., 2022a] and OmegaPLM [ Wu et al., 2022a]) both programs employ a geometry based module before feeding into the AF2 structure module. Due to this similar architecture of OF and RGN2, we would expect the performance of OF and RGN2 on structure prediction of orphans to improve in a similar manner in comparison to AF2. We assume that the overlap of training and chosen test set in the RGN2 study might have led to an overestimation of the accuracy for both RGN2 and AF2 on the respective set of orphans. According to its original study [ Lin et al., 2022a], ESMfold performed less accurately than AF2 on orphans and on proteins with an MSAdepth<10 and <100 . Nonetheless, one major advantage of pLM based predictors is speed. AF2 already decreased the runtime of predictions from formerly weeks on *ab initio* structure prediction servers (such as QUARK or Rosetta [ Xu and Zhang, 2012, Rohl et al., 2004]) to minutes. Yet, pLM based approaches promise to be multiple times faster than AF2 by skipping the computationally expensive MSA generation. Nevertheless, a language model would have to be retrained on high computing resources to stay up to date with continuously growing sequence and structure databases.

### Comparing structure predictions of *de novo* proteins and IDPs

For **AFGP** the presence of α -helices and high disorder is also approximated by experimental studies but none of the programs predicts the polyproline II-helices suggested by experiments [ Her et al., 2019]. One obstacle for structure prediction of highly dynamic proteins that becomes apparent here is the lack of prediction of ensembles of conformations. Absence of conformational ensembles is a general problem of predictions, experimental determination and deposits in the PDB [ Alderson et al., 2022, Saldaño et al., 2022, Lindorff-Larsen and Kragelund, 2021]. This could, for example, lead to a wrong estimation of disorder levels [ Ruff and Pappu, 2021]. Interestingly, for AFGP in *D. mawsoni*, AF2 is able to predict the experimentally confirmed structures [ Giubertoni et al., 2019]. AF2 predicts a polyproline II-helix for the peptide [ Giubertoni et al., 2019] and for the polypeptide the β -solenoid structure composed of T-(A/P)-A tandem repeats (see underlying data). This AFGP from *D. mawsoni* has not emerged *de novo* but from a serine protease, while being composed of the same repetitive structure as *de novo* emerged AFGP.

In the case of **Bsc4**, only the two pLM based programms (OF and ESMfold) are predicting the larger β -sheets determined by experiments [ Bungard et al., 2017] and both predict an α -helix around the same position as AF2 and RGN2 (K62-R83). In this case, a prediction with lower pLDDT might actually be closer to reality or reflect the conformational heterogenity arising from structural dynamics [ Del Alamo et al., 2022, Saldaño et al., 2022].

While it may be reassuring that all approaches predict the structure of **Goddard** effectively the same, the difference in pLDDT shows how pLDDT values may differ between programs while the underlying structure predictions do not.

For **NCYM**, the α -helix predicted by OF and AF2 are longer than determined by experiments (A46-G59) [ Matsuo et al., 2021]. The one α -helix predicted by RGN2 is shorter but in the correct position. The second α -helix predicted by OF is not supported by experimental data [ Matsuo et al., 2021]. RGN2 is the only method that predicts β -sheets which are also in the positions supported by experiments (R60-C64, C104-I107). Other β -sheets suggested by experiments were not predicted by any of the programs. This indicates that, when comparing the results of different structure prediction programs, the prediction with the highest confidence score is not necessarily the most suitable one.

Difference in pLDDT scoring while predicting both similar or different structures can become problematic. Especially, when pLLDT is used as a proxy metric in bulk studies [ Bruley et al., 2022, Wilson et al., 2022, Tunyasuvunakool et al., 2021, Akdel et al., 2022]. A switch in structure predictor would possibly lead to very different pLDDT values. Different programs could potentially predict the same structures while the pLDDT output is different as in the case for Goddard. As for AFGP, different pLDDT with the same predicted disorder can be obtained from different predictors. For Bsc4, only OF and ESMfold were capable of predicting β -sheets that were deduced experimentally, while for NCYM only RGN2 predicted correctly the experimentally confirmed β -sheets and the α -helix in the correct position. Only for Goddard all predicted structures were in agreement. While such an agreement did not apply to all pLM based approaches, all three were capable of predicting confirmed secondary elements that AF2 could not. In general, the selected *de novo* proteins with their structural heterogeneity, isolation in sequence space and disorder levels are a challenge for any prediction program. All predictions have an average low (70> pLDDT >50) to very low (<50 pLDDT) confidence. Such a low confidence can be an indicator of disorder and/or of low-quality MSAs [ Bordin et al., 2022]. Both disorder and low-quality MSAs are respectively a proposed property and a hallmark of *de novo* emerged proteins [ Bornberg-Bauer et al., 2021].

When comparing the structure predictions of *de novo* proteins to the ones of evolutionary conserved IDPs, the results indicate that the lower mean pLDDT for *de novo* protein predictions is not solely caused by high disorder levels ( Figure 4 & Table 2). Evolutionary conserved IDPs, which exhibit high disorder levels, still have a higher average pLDDT score for each prediction program (see Table 2). This higher mean pLDDT can be attributed to higher pLDDT for the secondary elements of the evolutionary conserved IDPs than for the secondary elements of *de novo* proteins. The findings also highlight that the prediction of secondary elements can be consistent among different prediction programs for conserved IDPs, while pLDDT varies significantly between programs, as it is also the case for *de novo* protein structure predictions ( Figures 4 & 3). The significantly higher pLDDT for predictions by AF2 for smaller proteins such as Goddard and Nop10 could be due to an easier global search problem for the energy minima of those smaller proteins.

### Implications of modern structure predictions for *de novo* proteins

The vast majority of all known proteins can be clustered into families, based on similarity of their folds, sequences and functions [ Chothia, 1992]. While members of these protein families presumably share ancestry, *de novo* proteins represent special cases as they do not seem to fit in any evolutionary established family. Each protein family or class of folds can be seen as small islands in a vast ocean of viable sequences and proteins [ Tretyachenko et al., 2022]. Only few of these islands have surfaced during the course of evolution while most remained submerged or plunged. *De novo* proteins can be seen as new islands, mutationally distant from all other islands in this ocean of sequences and could therefore provide unique structures and folds. Completely unique structures could further confirm *de novo* status of proteins for which no homologous sequences can be found in closely related genomes, since structure is more conserved than sequence [ Illergård et al., 2009]. Also, entirely new structures are highly unlikely to derive from an ancestral protein homologous in sequence but structurally different [ Illergård et al., 2009; Chothia and Lesk, 1986]. Novel folds were also rarely identified within new experimentally solved structures [ Tóth-Petróczy and Tawfik, 2014] but recent advancements will increase dramatically the structural coverage of the known sequence space and could lead to identification and definition of new protein folds and families [ Liu et al., 2022, Varadi et al., 2021, Bordin et al., 2023]. These advancements also provide new opportunities to search for structural homology of *de novo* proteins on a larger set of protein structures with popular structure homology algorithms already including predictions [ van Kempen et al., 2022; La et al., 2009; Holm, 2022; Aderinwale et al., 2022]. While we share the general enthusiasm of these recent advancements, it remains to be decided which structure predictors will be most suitable for *de novo* proteins. Eventually, accuracy can only be confirmed when *de novo* protein structures are solved experimentally, ideally with NMR [ Eicholt et al., 2022]. A key issue here is the unknown and potentially very large mutational distance of *de novo* proteins to the “islands” of protein families, *i.e.* the known realm within the vast sequence space. This accounts for any structure prediction approach, whether MSA or pLM based. Structures will only be predicted reliably if the sequences in training sets are close enough in sequence space to *de novo* proteins. Also, leveraging machine learning approaches for MSA generation could in general improve predictions for proteins with only remote partial homology to others [ Llinares-López et al., 2022; Petti et al., 2022]. Vice versa, such advancements in homology search for structure predictions could be employed for improved detection and confirmation of *de novo* emerged proteins. Additionally, it should be kept in mind that pLDDT, when used as a proxy metric for bulk analysis, can vary drastically between the different programs ( Figure 3) and is not practical to compare the actual confidence of different structure prediction programs to each other. Finally, the differences seen here between structure predictions and experimental approximations indicate that a decision for which predictors to use has to be made on a case-by-case basis for every *de novo* protein. Modular, open-source architectures such as OpenFold [ Ahdritz et al., 2022] might allow better customization and help deciding which model is the most useful for *de novo* proteins. Also, multiple models using MSA and pLM could be combined to obtain larger sampling of sequences. Yet without further experimental structure determination, in the words of George E. P. Box, “all models are wrong, but some are useful” [ Box, 1976].

## Data Availability

Zenodo: Assesing structure and disorder predictions tools for de novo emerged proteins in the age of machine learning https://doi.org/10.5281/zenodo.7615407. This project contains the following underling data:
• afgp_AF2.pdb (prediction of AFGP by AF2) • afgp_ESM.pdb (prediction of AFGP by ESMfold) • afgp_OF.pdb (prediction of AFGP by OF) • afgp_plddt.csv (list of pLDDT for each residue of each prediction of AFGP) •
AFGP_polyprotein_antarctic_cod.pdb (prediction of AFGP (polyprotein) from antarctic cod by AF2) • afgp_rgn2.pdb (prediction of AFGP by RGN2) •
Antifreeze_glycopeptide_antarctic_cod.pdb (prediction of AFGP (peptide) from antarctic cod by AF2) • bsc4_AF2.pdb (prediction of Bsc4 by AF2) •
bsc4_AF2_ranked2.pdb (prediction (2nd highest ranked of Bsc4 by AF2) • bsc4_ESMfold.pdb (prediction of Bsc4 by ESMfold) • bsc4_OF.pdb (prediction of Bsc4 by OF) • bsc4_plddt.csv (list of pLDDT for each residue of each prediction of Bsc4) • bsc4_RGN2.pdb (prediction of Bsc4 by RGN2) • disorder_lineplots.pdf (lineplots of disorder predictions) • disorder_values.csv (list of disorder values for each prediction of each protein) • gdrd_AF2.pdb (prediction of Goddard by AF2) • gdrd_ESMfold.pdb (prediction of Goddard by ESMfold) • gdrd_OF.pdb (prediction of Goddard by OF) • gdrd_plddt.csv (list of pLDDT for each residue of each prediction of Goddard) • gdrd_RGN2.pdb (prediction of Goddard by RGN2) • ncym_AF2.pdb (prediction of ncym by AF2) • ncym_OF.pdb (prediction of ncym by OF) • ncym_plddt.csv (list of pLDDT for each residue of each prediction of ncym) • ncym_RGN2.pdb (prediction of ncym by RGN2) • nycm_ESMfold.pdb (prediction of ncym by ESMfold) • p-values_all.csv (p-values of all statistical analyses) • denovo_sequences.fasta (amino acid sequences of analysed *de novo* proteins) • 1Y2Y_AF2.pdb (prediction of Nop10 by AF2) • 1Y2Y_OF.pdb (prediction of Nop10 by OF) • 1Y2Y_plddt.csv (list of pLDDT for each residue of each prediction of Nop10) • 1Y2Y_RGN2.pdb (prediction of Nop10 by RGN2) • 1Y2Y_ESMfold.pdb (prediction of Nop10 by ESMfold) • 2LM0_AF2.pdb (prediction of AF9 by AF2) • 2LM0_OF.pdb (prediction of AF9 by OF) • 2LM0_plddt.csv (list of pLDDT for each residue of each prediction of AF9) • 2LM0_RGN2.pdb (prediction of AF9 by RGN2) • 2LM0_ESMfold.pdb (prediction of AF9 by ESMfold) •
alpha_synuclein_AF2.pdb (prediction of alphasynuclein by AF2) •
alpha_synuclein_OF.pdb (prediction of alphasynuclein by OF) •
alpha_synuclein_plddt.csv (list of pLDDT for each residue of each prediction of alphasynuclein) •
alpha_synuclein_RGN2.pdb (prediction of alphasynuclein by RGN2) •
alpha_synuclein_ESMfold.pdb (prediction of alphasynuclein by ESMfold) • p53_AF2.pdb (prediction of p53 by AF2) • p53_OF.pdb (prediction of p53 by OF) • p53_plddt.csv (list of pLDDT for each residue of each prediction of p53) • p53_RGN2.pdb (prediction of p53 by RGN2) • p53_ESMfold.pdb (prediction of p53 by ESMfold) • globular_controls.fasta (sequences of globular controls) • idp_controls.fasta (sequences of disordered controls) • mean_plddt.ods (Mean values of pLDDTs) SRQR checklist for “Assessing structure and disorder prediction tools for *de novo* emerged proteins in the age of machine learning are deposited on Zenodo”: https://doi.org/10.5281/zenodo.7615407
-
Aubel_SRQR_checklist.pdf. Data are available under the terms of the Creative Commons Zero “No rights reserved” data waiver (CC0 1.0 Public domain dedication). All scripts and code used are deposited on Zenodo: https://doi.org/10.5281/zenodo.7615407.
• plddt_plotting.py (python script for plotting of pLDDT values) • count_aa.py (python script to count amino acids in multiline fasta file) •
R_stats_plots.txt (Code used in R Studio to perform statistical tests and plot disorder values) •
af2_palma_sbatch.sh
