# Supplementary material for: Assessing structure and disorder prediction tools for de novo emerged proteins in the age of machine learning
Source: F1000Res. 2023 Mar 29;12:347. [Version 1] doi: 10.12688/f1000research.130443.1 (PMC10126731; doi:10.12688/f1000research.130443.1)
Supplement: Peer review history

# Peer review: Reviewer response for version 1

**DOI:** 10.5256/f1000research.143204.r168292

**Published:** 2023-04-24

**Authors:** Isabelle Callebaut

**Affiliations:** 1 Muséum National d'Histoire Naturelle, UMR CNRS 7590, Institut de Minéralogie, de Physique des Matériaux et de Cosmochimie, IMPMC, Sorbonne Universite, Paris, Île-de-France, France

This paper is devoted to the evaluation of disorder and structure prediction methods for the specific case of *de novo *proteins. Structure prediction for such proteins is a challenge since, by definition, they have no homologs, on which AI-based prediction methods such as AlphaFold2 are based. Therefore, the authors examined recently developed natural language models of proteins, used for alignment-free structure predictions, to assess their value compared to those based on taking homologs into account. They performed a comprehensive comparison of the methods, based on the consideration of four *de novo *proteins whose structures were studied experimentally and carefully evaluated the influence of the chosen parameters.

 The result is a critical, thorough and well-argued analysis and discussion of the information provided by these methods and their limitations, which on the one hand provides a solid basis for anyone wishing to apply them to such cases and on the other hand highlights the complexity of the issue and the need for further development.

 My only small suggestion would be the following: the authors mainly discuss global features through the calculation of the mean probability for disorder, the fraction of disordered residues and the mean of the pLDDT values, even though the values per amino acid are displayed at the level of each figure (with more or less dispersion reflecting the heterogeneity of the proteins in term of structural properties). In my opinion, it would be interesting to compare, at the level of each amino acid of the 4 proteins considered, the disorder and pLDDT values. Indeed, as it has been rightly commented in the discussion, low pLDDT values are not necessarily associated with disorder, but with an inability of structure prediction methods to predict order (an hypothesis that is all the more plausible here in the case of *de novo* proteins). A comparison of the predictions of disorder and pLDDTs at the amino acid level would therefore perhaps qualify and complete the analysis, by highlighting the potentiality of hidden or conditional order. This point deserves at least a little more discussion, if not exploration.

Is the work clearly and accurately presented and does it cite the current literature?

Yes

If applicable, is the statistical analysis and its interpretation appropriate?

Yes

Are all the source data underlying the results available to ensure full reproducibility?

Yes

Is the study design appropriate and is the work technically sound?

Yes

Are the conclusions drawn adequately supported by the results?

Yes

Are sufficient details of methods and analysis provided to allow replication by others?

Yes

Reviewer Expertise:

Structural bioinformatics

I confirm that I have read this submission and believe that I have an appropriate level of expertise to confirm that it is of an acceptable scientific standard.

# Peer review: Reviewer response for version 1

**DOI:** 10.5256/f1000research.143204.r168293

**Published:** 2023-04-21

**Authors:** Caroline Weisman

**Affiliations:** 1 Lewis-Sigler Institute for Quantitative Genomics, Princeton University, Princeton, New Jersey, USA

**General comments**

 The aim of this paper is to assess the suitability of modern protein structure prediction methods for use on orphan and *de novo* proteins. If structure prediction methods could reliably be used for these proteins, much headway could be made in generating hypotheses as to their functions and possible origins, via computationally assessing whether their structures match those of any known, conserved proteins. As this work from these authors and others has shown, experimental characterization, both functional and structural, of this class of proteins is difficult, and so computational characterization is especially valuable.

 There are several reasons to worry that structure prediction tools may not be well-suited for these proteins, and therefore to think that a direct test of the issue is essential. There is a general concern that, because they are relatively rare among proteins and not often the subject of primary thrusts of biological interest, they have not been much included in standard benchmarking datasets for these tools. Combined with the strong possibility that structural or other features of these proteins relevant to the structure prediction methods may be different than those of conserved proteins, this motivates a worry that methods benchmarked on conserved proteins will not generalize well to this class of proteins. There is also a much more specific and acute concern motivated by the methodology of a subset of these tools: as the authors note, tools like AlphaFold use alignments comprised of homologous sequences of the proteins, which are definitionally unavailable or highly restricted for orphans and *de novo* proteins. It is thus entirely unclear whether structure prediction methods will make correct predictions for orphan and *de novo* proteins.

 The goal of this paper is therefore extremely well-motivated and of broad general interest for the field. Before we rush to apply these methods and take their results at face value, as is already happening, we should first pause to assess whether their results are at all accurate. I am grateful to the authors for having tackled the issue here.

 The authors take the general approach of using the small number of these proteins for which there is some amount of convincing experimental evidence regarding their structure, and performing a careful, small-N case study to assess two things: a) how well the structures of these proteins predicted by these tools line up with the experimental reality; and b) to what extent the predicted structures from these tools agree with one another. This seems to be a well-motivated and useful way of getting at the issue.

 Most of my suggestions concern the organization of the manuscript, as I think that the results are actually more important than the current structure allows one to easily see at present. I found the paper difficult to follow due to its organization and to the particular results that were presented (and if I have misunderstood the aims themselves, clarification in the text would be useful). I also wonder about including some additional comparisons or analyses that strike me as relevant to these central questions and that could be easily produced from the data that already exist in the manuscript.

**Suggestions for reorganization**

 I found the flow of results in the manuscript to be a little confusing and scattered. It is not clear to me what the overall organizational paradigm in their presentation is; I felt somehow pulled back and forth. Clarification here would help.

 A suggestion for a structure that would have worked better for me is to divide the manuscript into two parts, corresponding to the a) and b) aims that I described above. So, for example, Figure 1, which compares the results of the disorder prediction methods to one another, would fall into a section corresponding to a); and Figure 2, which compares predictions to experimental ground truths, would fall into b); Figures 3 and 4, which compare methods to one another, into a).

**Highlighting central analyses by moving from the Discussion to Results and adding figures**

 This organization makes salient what I feel to be two missing analyses that could be produced from the results already in the manuscript.

 First, to me, the most important analysis is the comparison of the existing experimental structure data for the *de novo*/orphan proteins to the structures predicted by these programs. As the authors note, these existing data are not fully-solved structures, so that a traditional e.g., RMSD/visual overlay analysis cannot be performed. Nonetheless, qualitative, heuristic comparisons can and should be done. Indeed, the authors do this in the Discussion section. But I think this should be moved to Results under its own heading: it is arguably the key result of the paper. Perhaps the authors are trying to be conservative in calling it a Result, as it is qualitative and therefore perhaps feels subjective – but I disagree! One could systematize this analysis to some extent by e.g., making a table to systematically carry out the qualitative comparisons that they describe in the text. This kind of analysis is what I expect upon reading the title and abstract of this paper, and so I feel strongly that to bury it in the Discussion section with little fanfare does not do it justice. Similarly, some kind of overall summary – the punch line of, in their estimation, how well these structures have been predicted – would be useful. (A change in the title to this effect to describe the actual results of the assessment could constitute this.)

 Second, I am not sure why Figure 2 does not include results for predicted and experimentally characterized disorder content for the *de novo*/orphan genes. This is another result that is described verbally in the text of the Discussion, but for the same reasons as above strikes me as a key result, and could easily be included on the axes of Figure 2.

**Adding a summary/bottom line**

 The authors may again be trying to be conservative and not overstate conclusions from their analyses, as is their prerogative. But I would like to note that, as a reader, I do not feel that an overall summary or punch line would have been misplaced. There are hints of this, but they are again buried in the Discussion; for example, the sentence *“When comparing these single disorder values for the de novo proteins at hand, only results from the fraction of residues in a disordered region predicted by flDPnn correspond to the experimental data” *strikes me as an enormously important conclusion, but is nowhere in the Results, is not emphasized beyond this one-off comment, and so could easily be missed by the casual reader. I think this kind of telegraphic conclusion is important for the results being recognized and taken up by the field.

 The authors have done much work to benchmark these methods. Some kind of summary statement in answer to the actual question motivating the work -- how well do these methods perform on *de novo* protein structures? -- feels apt and missing. The title of the MS would be one place to send this message. A dedicated section in the Results containing conclusions like the one above would be another. 

 Similarly, the finding about parameter sensitivity for disorder is extremely important and addresses a major issue in the field: this parameter is often different between studies and is sometimes not even reported. This is an important call to attention for the field. The analysis of which metrics for agreement or disagreement are reported and how they lead to very different conclusions is extremely important for the same reason. This result is more clearly articulated in the Results, but also bears repeating in a section like the one proposed.

**Additional questions/comments**

 Following are some more minor questions about the scientific content and analyses in the text.

- The MS says, and this strikes me as correct, that pLDDT should not be compared between prediction methods – but much of the paper seems to do just that (the final paragraph before discussion and the whole right panel of Figures 3 and 4). I am confused as to the apparent contradiction. Similarly, is it meaningful to say that there are fewer significant differences in pLDDT for conserved IDPs than *de novo* genes? Is the meaningful comparison not something about the similarity or differences between the predicted structures themselves (left panel of Figures 3 and 4)?
- Why does the order of disorderedness in a set of proteins (which protein has most vs least) matter? I’m not sure this is a useful metric to be reporting. There is a reference to previous literature, but it does not explain why this is an informative metric.
- If the suggested plot of predicted vs observed disorder for *de novo*/orphan proteins (like Figure 2) were included, there may be a clear answer to a question that emerges: the data suggest pretty strongly that disorder prediction programs underestimate disorder in highly-disordered conserved proteins, and that they underestimate disorder in *de novo* proteins. Is the underestimation in *de novo* proteins merely due to their high disorder, or is their lack of conservation also contributing? If the quantitative degree of difference is similar to the difference for conserved proteins, the former seems likely; if it is worse, the latter seems likely. This would be useful to know.
- On parameter sensitivity: insofar as IUpred has only one parameter, it seems possible to dig in a little more meaningfully. Can you say something (not necessarily an experiment; just a comment) about the use of short vs long in IUpred? What does that parameter mean? What are they intended to represent? When is one more appropriate than the other per the manual? Do you find that one agrees more with the experimental data?
- On disordered residues: the comparison between average disorder and percent of disordered residues is important and well-taken, but in terms of predicting the structure, is it useful to also consider the physical distribution of disordered residues ( *which *residues are disordered, and whether methods agree)? This seems like an important metric to me in assessing the agreement between methods. For example, in an extreme case, two methods could each predict that 50% of residues in a protein are disordered, but have 100% disagreement about *which* residues are disordered if they predict nonoverlapping sets of residues to be disordered. In these types of scenarios, the percentage alone may be a misleading indicator of agreement.

Is the work clearly and accurately presented and does it cite the current literature?

Partly

If applicable, is the statistical analysis and its interpretation appropriate?

Yes

Are all the source data underlying the results available to ensure full reproducibility?

Yes

Is the study design appropriate and is the work technically sound?

Yes

Are the conclusions drawn adequately supported by the results?

Yes

Are sufficient details of methods and analysis provided to allow replication by others?

Yes

Reviewer Expertise:

De novo genes, computational genomics. No expertise in structure prediction.

I confirm that I have read this submission and believe that I have an appropriate level of expertise to confirm that it is of an acceptable scientific standard.
